# Proteomic Profiling of Hu Sheep Placental Development Across Gestational Stages Reveals Stage-Specific Regulatory Networks

**DOI:** 10.3390/ijms26094236

**Published:** 2025-04-29

**Authors:** Zhibo Wang, Jiahe Guo, Tianning Dong, Yaxu Liang, Zhipeng Liu, Feng Wang, Yanli Zhang

**Affiliations:** 1 Jiangsu Livestock Embryo Engineering Laboratory, College of Animal Science and Technology, Nanjing Agricultural University, Nanjing 210095, China; 2020205010@stu.njau.edu.cn (Z.W.); gjh18752783920@163.com (J.G.); 2023105042@stu.njau.edu.cn (T.D.); 2021205036@stu.njau.edu.cn (Y.L.); liuzhipeng0929@163.com (Z.L.); caeet@njau.edu.cn (F.W.); 2 Shanghai Animal Disease Prevention and Control Center, Shanghai 201103, China

**Keywords:** placental development, gestational stages, proteomics, sheep, cell proliferation, nutrient transport

## Abstract

Placental development plays a pivotal role in ensuring successful pregnancy outcomes, yet its molecular regulatory mechanisms in sheep remain poorly characterized. This study aimed to systematically investigate stage-specific proteomic dynamics and functional adaptations in ovine placental tissues across gestation to elucidate molecular drivers of placental maturation. Using data-independent acquisition proteomics, we identified 7774 proteins in Hu sheep placental tissues at gestational days 50, 80, and 120. Comparative analysis revealed 1450, 1026, and 1964 differentially expressed proteins (DEPs) in the 50 d vs. 80 d, 80 d vs. 120 d, and 50 d vs. 120 d comparisons, respectively. DEPs were functionally enriched in biological processes including cell proliferation, apoptosis, angiogenesis, nutrient transport, and steroid synthesis, with prominent involvement of the PI3K-Akt, MAPK, and estrogen signaling pathways. Protein interaction networks identified SRC, MAP3K1, KRAS, and TJP1 as central regulators exhibiting dynamic expression patterns across gestation. Temporal expression trends showed progressive upregulation of tight junction, immune response, and glucose metabolism proteins, contrasting with downregulation of endoplasmic reticulum protein processing and proteasome components. Validation experiments confirmed elevated proliferation/transport gene expression at 80 d versus 50 d, followed by increased apoptosis/transport genes and decreased proliferation markers at 120 d. This comprehensive proteomic profiling reveals stage-specific regulatory networks governing placental development in sheep, highlighting coordinated shifts in proliferative, metabolic, and structural remodeling processes. These findings advance our understanding of placental adaptation mechanisms and provide valuable insights for improving reproductive management in livestock species.

## 1. Introduction

Reproductive efficiency in ruminants serves as a critical indicator for both economic viability and sustainable development in animal husbandry [1,2]. Studies report that despite optimal herd management, high rates of pregnancy loss persist in both sheep and cattle populations, representing a major constraint on reproductive efficiency [1,3]. Placental development during gestation significantly impacts ovine reproductive performance. As a unique exchange organ between dam and fetus originating from extraembryonic tissues, the placenta fulfills multiple essential functions throughout pregnancy, including facilitating maternal physiological adaptation, regulating fetal immune acceptance, and providing nutritional support [4,5]. Protein expression patterns in the placenta demonstrate marked dynamic changes during gestation, reflecting cellular and tissue-level functional adaptations [5]. Given proteins’ central role in biological processes, investigating gestational stage-specific protein expression differences in ovine placenta could reveal molecular mechanisms underlying placental development and adaptive responses.

Hu sheep, valued for their year-round estrus, high fecundity, and adaptability to intensive housing, are China’s predominant maternal breed in meat sheep production systems [6]. Elucidating the molecular genetic mechanisms underlying Hu sheep’s prolificacy holds significant importance. Previous research has predominantly focused on placental morphological and histological characteristics, with limited exploration of dynamic proteomic changes during gestation [7]. Advances in proteomic technologies now enable comprehensive profiling of placental protein expression, offering molecular insights into complex biochemical processes that complement genomic and transcriptomic studies [8,9]. During mid-to-late gestation, Hu sheep face substantial nutritional stress, elevating scientific interest in placental function as the critical mediator of maternal–fetal nutrient exchange [7]. Despite previous studies, a systematic characterization of temporal proteomic signatures in the ovine placenta across developmental stages is lacking. Investigating stage-specific placental protein expression could unravel physiological adaptations in pregnancy, informing strategies to enhance reproductive efficiency. Understanding these regulatory mechanisms may optimize breeding management practices, potentially improving conception rates and lamb survival outcomes.

Therefore, this study investigated Hu sheep placental samples collected after 50 d, 80 d, and 120 d of gestation using data-independent acquisition (DIA) proteomics combined with bioinformatics analysis. The experimental design systematically identified candidate differentially expressed proteins (DEPs) or target molecules associated with placental development, hormonal secretion, and nutrient transport functions in ovine placentas.

## 2. Results

### 2.1. Differential Protein Abundance Analysis Across Ovine Gestational Stages

**Figure 1:**
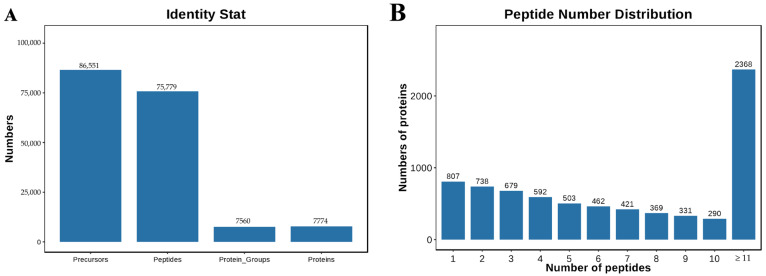
Quality assessment of DIA data for sheep placenta at different gestation periods. (**A**) Under specific FDR filtering criteria, the DIA data for sheep placenta identified precursors, peptides, proteomes, and proteins. (**B**) The analysis of peptide distribution results shows the distribution of protein quantities corresponding to different numbers of peptides.

Data-independent acquisition (DIA) mass spectrometry analysis of placental samples from different ovine gestational stages identified 86,551 precursors, 75,779 peptides, 7560 protein groups, and 7774 proteins (Figure 1A). Peptide distribution analysis demonstrated that 2368 proteins were identified with ≥11 peptides, 807 proteins with a single peptide, and the remaining proteins with 2–10 peptides (Figure 1B), confirming high data coverage and reliability for subsequent differential expression analysis.

**Figure 2:**
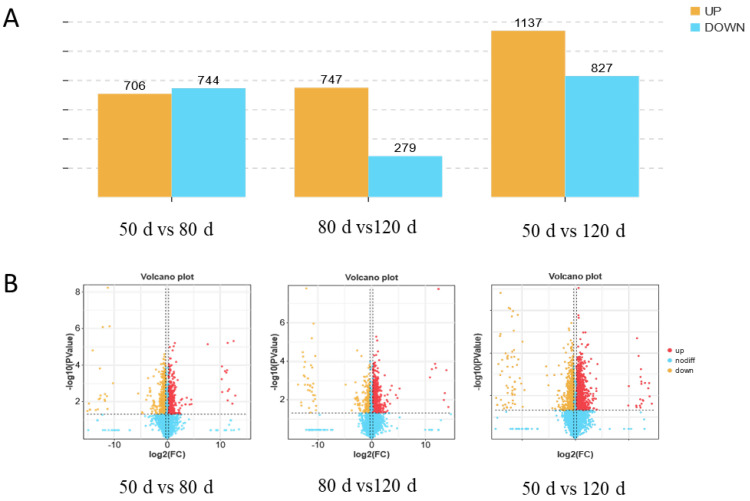
Characteristics of the placental proteome in sheep at different stages of pregnancy. (**A**) Number of differentially expressed proteins in the placenta of three sheep groups. (**B**) Volcano plots of differentially expressed proteins in the three sheep groups.

Comparative analysis revealed significant temporal changes in protein abundance. Between 50 d and 80 d of gestation, 706 proteins were significantly upregulated and 744 were downregulated. From 80 d to 120 d, 747 proteins exhibited upregulation compared to 279 downregulated proteins. Notably, comparing early (50 d) and late (120 d) gestation identified 1137 upregulated and 827 downregulated proteins (Figure 2), highlighting dynamic proteomic remodeling throughout pregnancy. The complete list of differential proteins is presented in the Supplementary Excel file.

### 2.2. GO Annotation and Enrichment Analysis of Differentially Abundant Proteins in Ovine Placenta Across Gestational Stages

**Figure 3:**
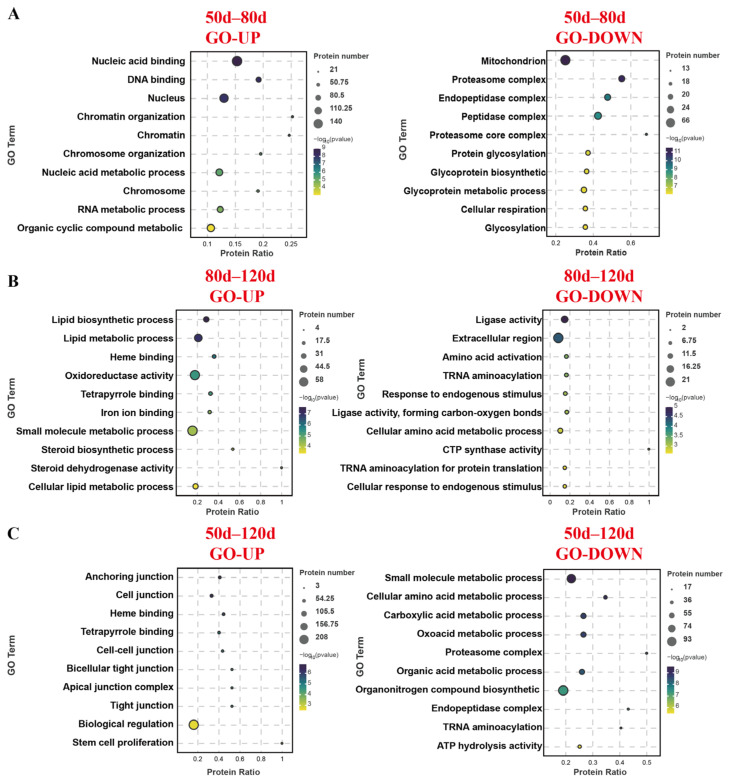
GO functional enrichment of differentially expressed proteins in the placenta of three sheep groups. (**A**) GO analysis of 50–80 d differential proteins. (**B**) GO analysis of 80–120 d differential proteins. (**C**) GO analysis of 50–120 d differential proteins.

GO analysis of upregulated proteins during the 50–80 d gestational period revealed enrichment in biological functions related to nucleic acid binding, transcription/translation, and organic metabolism, while downregulated proteins were predominantly associated with mitochondrial activity, cellular respiration, glycosylation, and protease synthesis/metabolism (Figure 3). In the 80–120 d comparison, upregulated proteins showed functional enrichment in lipid metabolism, small molecule metabolism, iron ion binding, and steroid synthesis, whereas downregulated proteins were linked to amino acid metabolism, ligase activity, response to endogenous stimuli, and tRNA aminoacylation. Notably, the 50–120 d gestational comparison demonstrated upregulated protein enrichment in cell junction organization, biological regulation, and stem cell proliferation, contrasting with downregulated proteins enriched in small molecule metabolism, amino acid metabolism, protease synthesis, tRNA aminoacylation, and ATP catabolic activity.

### 2.3. KEGG Annotation and Enrichment Analysis of Differentially Abundant Proteins in Ovine Placenta Across Gestational Stages

**Figure 4:**
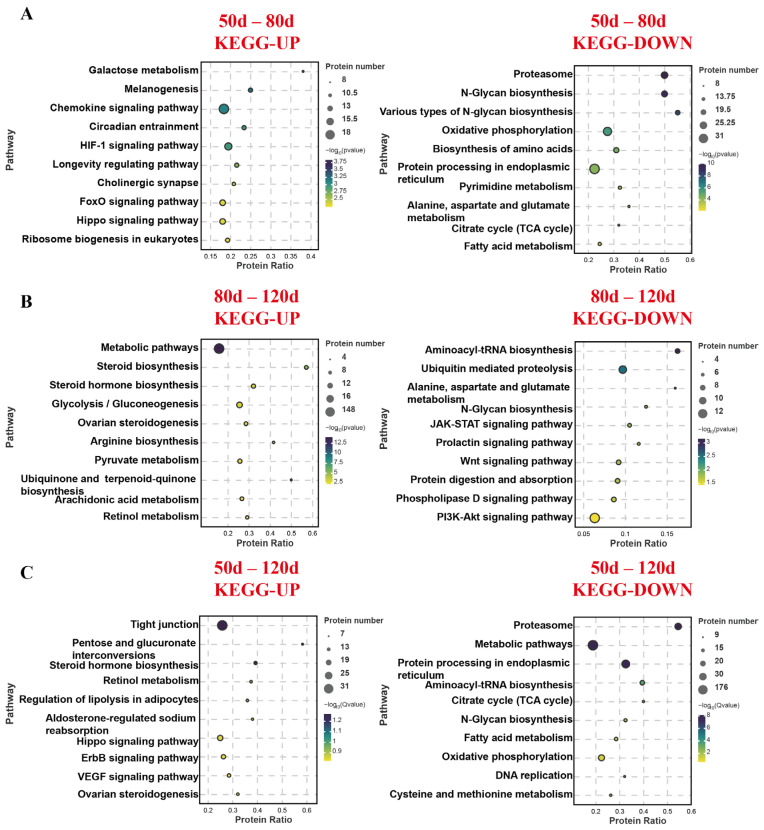
KEGG pathway enrichment of differentially expressed proteins in the placenta of three sheep groups. (**A**) KEGG analysis of 50–80 d differential proteins. (**B**) KEGG analysis of 80–120 d differential proteins. (**C**) KEGG analysis of 50–120 d differential proteins.

KEGG pathway analysis (Figure 4) revealed distinct functional patterns across gestational stages. During the 50–80 d period, upregulated proteins in placental cotyledons were primarily enriched in nutrient metabolism-related pathways such as carbohydrate metabolism, steroid hormone synthesis, and amino acid metabolism, whereas downregulated proteins were associated with amino acid metabolism, N-glycan biosynthesis, TCA cycle, and fatty acid metabolism. In the 80–120 d comparison, upregulated proteins again showed enrichment in carbohydrate metabolism, steroid hormone synthesis, and amino acid metabolism, while downregulated proteins were linked to amino acid metabolism, N-glycan biosynthesis, JAK-STAT signaling, Wnt signaling, prolactin signaling, and protein metabolic pathways. Analysis of the full gestational span (50–120 d) demonstrated upregulated protein enrichment in tight junction formation, carbohydrate/lipid/amino acid metabolism, and proliferation-related pathways (Hippo/YAP, ErbB, VEGF), contrasting with downregulated proteins involved in amino acid metabolism, N-glycan biosynthesis, TCA cycle, and fatty acid metabolism.

### 2.4. Temporal Expression Profiling of Differentially Abundant Placental Proteins

**Figure 5:**
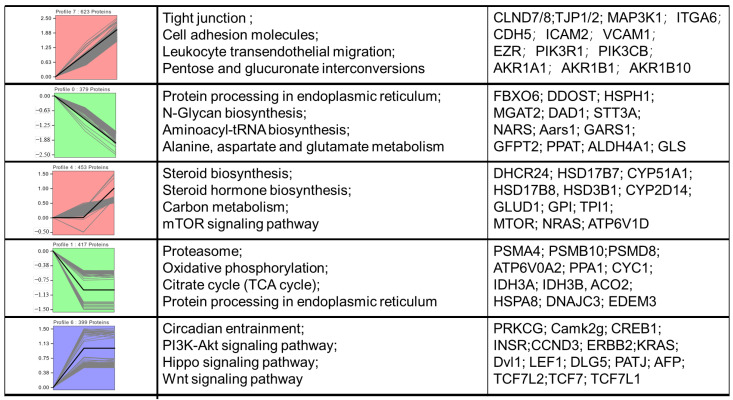
Dynamic expression patterns of proteins during placenta development. Note: Clusters of differentially expressed proteins in pituitary. Corresponding biological processes are shown next to each cluster.

To characterize expression patterns of differentially abundant proteins (DEPs) across three placental developmental phases, we performed k-means clustering of all placental DEPs, partitioning them into five distinct clusters (Figure 5). These clusters exhibited stage-specific expression trajectories and distinct biological functional enrichments. Cluster1 DEPs demonstrated progressive upregulation across developmental stages, with significant enrichment in tight junction assembly, immune responses, and carbohydrate metabolism. In contrast, Cluster2 proteins showed gradual downregulation throughout gestation, enriched in endoplasmic reticulum protein processing, N-glycan biosynthesis, and metabolic pathways. Cluster3 exhibited marked upregulation specifically during the 80–120 d phase (non-significant at 50–80 d), with functional enrichment in steroid biosynthesis and mTOR signaling pathways. Cluster4 proteins were significantly downregulated during 50–80 d (non-significant thereafter), primarily associated with proteasome function, energy metabolism, and endoplasmic reticulum protein processing. Cluster5 demonstrated significant upregulation at 50–80 d (non-significant in later stages), enriched in necroptosis regulation, PI3K-Akt signaling, and Hippo pathway activation.

### 2.5. Identification of Core Regulatory Proteins in Ovine Placenta Across Gestational Stages

**Figure 6:**
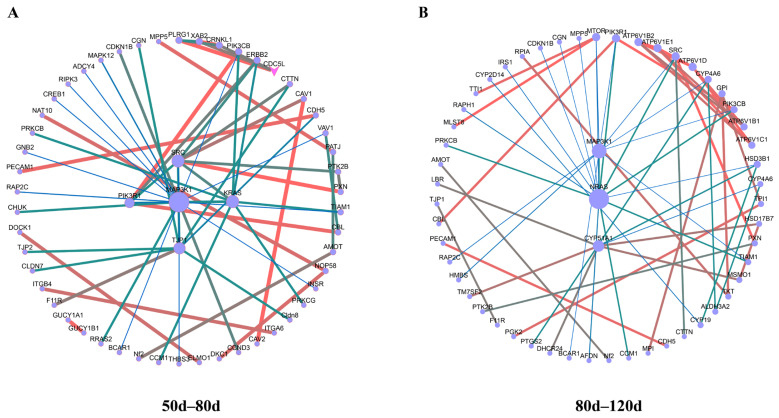
Hup network construction among different stages. (**A**): Hup network construction in 50–80 d. (**B**): Hup network construction in 80–120 d.

Protein–protein interaction (PPI) network analysis of placental cotyledon DEPs across gestational stages (50 d, 80 d, and 120 d) was conducted using Cytoscape 3.10.0, with key subnetworks extracted via the MCODE algorithm (Figure 6).

During the 50–80 d gestational phase, a central regulatory network emerged, comprising SRC, MAP3K1, KRAS, TJP1, and PIK3R1. SRC (proto-oncogene tyrosine-protein kinase Src), a non-receptor tyrosine kinase, critically regulates cellular proliferation, migration, and survival. MAP3K1 (mitogen-activated protein kinase kinase kinase 1) acts upstream in MAPK signaling pathways, modulating stress responses, proliferation, and apoptosis. KRAS (Kirsten rat sarcoma viral oncogene homolog), a pivotal Ras family member, coordinates signal transduction and mitogenic regulation while bridging MAPK pathway activation. TJP1 (tight junction protein 1), an essential component of intercellular junctions, maintains epithelial barrier integrity and cell polarity. PIK3R1 (phosphoinositide-3-kinase regulatory subunit 1) regulates PI3K-mediated cellular growth, proliferation, and metabolic reprogramming. This tightly interconnected network suggests synergistic coordination of placental development and functional remodeling during this critical gestational window.

### 2.6. Validation of Differentially Expressed mRNAs and MAPK Pathway Proteins in Ovine Placenta Across Gestational Stages

**Figure 7:**
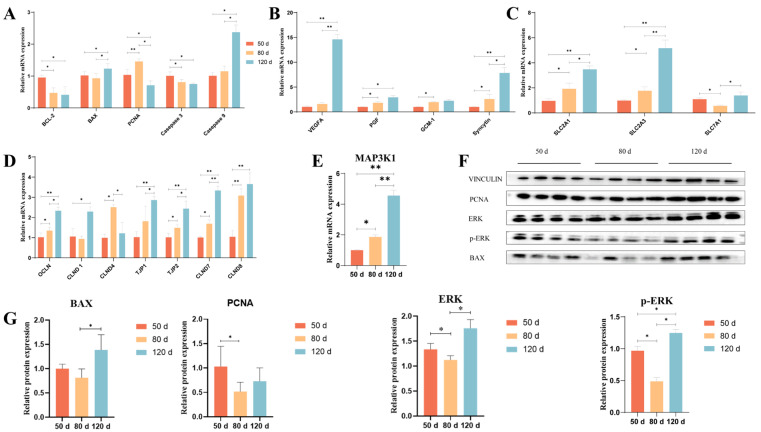
mRNA and MAPK pathway protein expression levels of placental differential function in sheep at different gestational stages. (**A**): The mRNA expression levels of genes related to cell proliferation and apoptosis. (**B**): The mRNA expression levels of genes related to the functions of placental trophoblast cells. (**C**): The mRNA expression levels of genes related to nutrient transport. (**D**): The mRNA expression levels of genes related to tight junction proteins. (**E**): The mRNA expression level of MAP3K1. (**F**): Western blot analysis of PCNA, BAX, ERK and P-ERK in ovine placenta across gestational stages. (**G**): Quantitation of the western blot results of Figure 7F. “*” or “**” indicates a statistically significant difference at the *p* < 0.05 or *p* < 0.01 level among 50 d, 80 d, and 120 d groups, respectively.

qRT-PCR and Western blot (WB) analyses validated stage-specific expression patterns of key mRNAs and proteins (Figure 7). Compared to 50 d, placental tissues at 80 d exhibited significant upregulation of proliferation-related PCNA, placental growth factors (PGF, GCM-1, Syncytin), glucose transporters (SLC2A1, SLC2A3), tight junction components (OCLN, CLDN4, CLDN7, CLDN8, TJP-2), and MAP3K1 mRNA levels, alongside downregulation of anti-apoptotic BCL-2, caspase3, and amino acid transporter SLC7A1. From 80 d to 120 d, pro-apoptotic BAX and caspase9, angiogenic VEGF, Syncytin, nutrient transporters (SLC2A1, SLC2A3, SLC7A1), junctional proteins (OCLN, TJP-1, TJP-2, CLDN7, CLDN8), and MAP3K1 mRNAs were upregulated, while PCNA and CLDN4 showed decreased expression. WB results (Figure 7F,G) confirmed reduced protein levels of PCNA, ERK, and phosphorylated ERK (p-ERK) at 80 d versus 50 d, followed by elevated BAX, PCNA, ERK, and p-ERK levels at 120 d compared to 80 d.

## 3. Discussion

The placenta functions as a dynamic endocrine organ, secreting numerous factors into maternal circulation to support fetal development. However, understanding placental development remains challenging due to limited access to early-stage placental tissues and the lack of reliable long-term in vitro or animal models [10]. Ruminant placentation exhibits unique adaptations distinct from other mammals, initially developing a non-invasive epitheliochorial structure during early gestation, followed by binucleate trophoblast cell migration and fusion with uterine luminal epithelium to form syncytial plaques in sheep [11]. This process facilitates placental attachment to endometrial caruncles, forming vascularized cotyledons critical for nutrient exchange [1,12]. While most studies focus on early gestation and embryo attachment, proteomic changes during mid-to-late placental development remain poorly characterized. Our study addressed this gap by employing data-independent acquisition (DIA) proteomics to analyze ovine placental cotyledons after 50, 80, and 120 days of gestation. The results demonstrated extensive proteome remodeling across developmental stages, with 1350 differentially expressed proteins (DEPs) identified between 50 and 80 days, 1026 DEPs between 80 and 120 days, and 1964 DEPs when comparing early (50 d) and late (120 d) gestation. These findings underscore the placenta’s dynamic molecular adaptations to meet evolving fetal nutritional demands during mid-to-late pregnancy.

The syncytiotrophoblast layer of the placenta serves as both a functional barrier and a critical interface for maternal–fetal nutrient exchange. This structure forms initially through the fusion of mononuclear cytotrophoblasts during implantation, with ongoing syncytialization throughout gestation to expand the surface area for nutrient transport [13]. TJP-1 (tight junction protein 1) localizes to intercellular boundaries between cytotrophoblasts and syncytiotrophoblasts, playing a conserved role in placental development across species [14]. In addition, there have been studies using gene knockout mouse models to determine the role of closure band proteins in early development [15]. Studies in knockout mice demonstrate that TJP-1 deficiency disrupts placental vascularization and chorioallantoic fusion [16], aligning with our findings of progressive TJP-1 upregulation in ovine placental cotyledons across gestation. Our proteomic analysis revealed increasing enrichment of tight junction pathway components in placental DEPs as pregnancy advanced. The rising TJP-1 expression likely reflects adaptive mechanisms to modulate vascular permeability and enhance nutrient transport efficiency [14,17], paralleled by sustained increases in CLDN7 and CLDN8—claudin family proteins that regulate paracellular permeability [18]. These proteins interact with TJP-1 to scaffold diverse junctional complexes [19], inversely correlating with tissue permeability [20]. Tight junction dynamics are hormonally modulated by progesterone, prolactin, and placental lactogens [21], with calcium signaling and kinase pathways further fine-tuning paracellular transport [22]. The coordinated upregulation of these components suggests a concerted effort to optimize placental barrier function during late gestation.

The placenta profoundly modifies maternal metabolism by secreting substantial quantities of hormones into maternal circulation [23], extending beyond its primary role in fetal nourishment. These placental-derived hormones orchestrate systemic maternal adaptations by regulating most maternal tissues and organs, thereby facilitating the maintenance of pregnancy, nutrient mobilization, parturition, and lactation. Concurrently, certain placental hormones are released into fetal circulation to regulate developmental processes, growth trajectories, and parturition timing [24,25]. Our findings align with previous reports demonstrating that maternal serum estradiol and estriol levels progressively increase throughout gestation, peaking during late pregnancy [26], while progesterone levels rapidly rise during early pregnancy and plateau mid-gestation before further increasing in late gestation [27]. The significant enrichment of differential proteins in steroid biosynthesis pathways during the 80–120 d gestational phase, as observed in our study, likely reflects the placenta’s intensified hormone production in late gestation, which serves as the principal source of maternal estrogens and progesterone [27]. These steroid hormones exhibit complementary regulatory functions: estrogens stimulate uterine expansion, enhance myometrial contractility, and promote mammary gland development, whereas progesterone suppresses premature uterine contractions and facilitates placental maturation while mitigating preterm labor risks [28].

Our study identified significant upregulation of key ERK/MAPK pathway components—NRAS, KRAS, and MAP3K1—during 50–80 d and 50–120 d gestational intervals. In mammals, ERK/MAPK activation is initiated by growth factor–receptor interactions, particularly tyrosine kinase receptors for FGF, EGF, and VEGF [29]. This pathway promotes trophoblast proliferation, survival, and establishment of the blood–placental barrier, which expands the exchange surface for nutrients and gases [30].

Comparative proteomic profiling of ovine placental cotyledons revealed dynamic remodeling of junctional complexes across gestation. The tight junction proteins TJP-1, CLDN7, and CLDN8 exhibited progressive upregulation, reflecting functional adaptations to enhance selective barrier integrity and maternal–fetal exchange efficiency. These proteins govern paracellular permeability and cell–cell adhesion, with elevated expression likely optimizing nutrient transport capacity during late gestation [18,19]. The coordinated regulation of ERK/MAPK signaling and junctional components underscores multifaceted mechanisms driving placental maturation to meet escalating fetal demands.

This study identified distinct proteomic profiles in ovine placental cotyledons across gestational stages, with differential expression of key proteins including TJP-1, CLDN7, CLDN8, KRAS, NRAS, and MAP3K1. KRAS and NRAS, critical members of the Ras family, and MAP3K1—an upstream regulator of the MAPK signaling cascade—exhibited stage-specific abundance changes. These proteins modulate cellular proliferation, differentiation, and apoptosis, suggesting their dynamic expression may be closely associated with placental growth and functional remodeling [29,30].

The progressive upregulation of TJP-1, CLDN7, and CLDN8 across gestation aligns with their roles in maintaining tight junction integrity and regulating paracellular permeability [18,19]. Such adaptations likely enhance nutrient transport efficiency while balancing barrier selectivity, as evidenced by concurrent ERK/MAPK pathway activation and vascularization-related protein shifts [17]. Furthermore, stage-specific alterations in signal transduction proteins highlight the placenta’s capacity to dynamically integrate hormonal and growth factor cues [21,22].

## 4. Conclusions

These findings provide a molecular framework for understanding placental adaptations to fetal demands, offering insights for optimizing reproductive management strategies in ewes. Future studies could build upon these findings to delineate mechanistic interactions between identified proteins and placental developmental milestones, particularly their roles in maternal–fetal crosstalk and nutrient allocation.

## 5. Materials and Methods

### 5.1. Experimental Animals and Tissue Collection

All experimental Hu sheep were obtained from Qianbao Animal Husbandry Co., Ltd. (Yancheng, China). High-prolificacy Hu sheep (2–3 years old, producing ≥3 lambs per parity consecutively over two pregnancies) were selected based on pedigree records. Following a synchronized estrus protocol, placental chorionic trophoblast tissues were aseptically collected at 50 d, 80 d, and 120 d of gestation (*n* = 3 per timepoint). Samples were immediately flash-frozen in liquid nitrogen (−196 °C) and stored at −80 °C. The experimental protocol was approved by the Nanjing Agricultural University Animal Welfare Ethics Committee (Approval No.: SYXK2022-0031).

### 5.2. Protein Extraction and Digestion

Procedures followed Zhang et al. [31] with modifications. Briefly, tissues were homogenized in lysis buffer (containing SDS, urea, and protease inhibitors). Homogenates were centrifuged at 12,000× *g* for 15 min at 4 °C, and supernatants were quantified using a BCA assay. Proteins were reduced with 10 mM DTT (56 °C, 30 min), alkylated with 55 mM iodoacetamide (25 °C, 30 min, dark), and precipitated overnight with pre-chilled acetone (−20 °C). Precipitates were washed twice with cold acetone, air-dried, and digested with sequencing-grade trypsin (1:50 *w*/*w*, 37 °C, 16 h). 

### 5.3. High-pH Liquid Chromatography Fractionation

The liquid chromatography system (UltiMate 3000, Thermo Fisher Scientific, Waltham, MA, USA) was interfaced with a timsTOF Pro2 mass spectrometer (Bruker Daltonics, Bremen, Germany) featuring ion mobility spectrometry–quadrupole time-of-flight (IMS-Q-TOF) technology. Chromatographic separation was performed using an Aurora Series AUR3-15075C18 column (IonOpticks, 15 cm × 75 μm ID, 1.7 μm particles, 120 Å pores) maintained at 50 °C with a constant flow rate of 400 nL/min. Following reconstitution in 0.1% formic acid (FA), 200 ng peptide samples were separated using a 60-min nonlinear gradient: the initial 4% mobile phase B (80% acetonitrile/0.1% FA) was increased to 28% over 25 min, followed by 44% in 10 min, 90% in 10 min (maintained for 7 min), and finally re-equilibrated at 4% for 8 min.

Data-independent acquisition (DIA) was conducted in diaPASEF mode with 22 precursor isolation windows (40 Th each) spanning m/z 349-1229. To optimize MS1 cycle time, we implemented a variable repetition scheme (2–5 steps) within the 13-scan diaPASEF acquisition framework. Collision energy during PASEF MS/MS scanning was linearly modulated based on ion mobility (1/K0), decreasing from 59 eV at 1.6 Vs/cm^2^ to 20 eV at 0.6 Vs/cm^2^. 

### 5.4. DIA: Nano-HPLC-MS/MS Analysis

Dried peptide samples were redissolved in 30 μL of solvent A (0.1% aqueous formic acid) and subjected to in-depth analysis using an online nano-electrospray liquid chromatography–tandem mass spectrometry (nano-LC-MS/MS) system on an Orbitrap Fusion Lumos Tribrid mass spectrometer. The EASY nLC 1200 ultra-high-performance liquid chromatography system was integrated to ensure analytical precision. During the experiment, 3 μL of sample was loaded onto the analytical column. A 120-min gradient elution program was applied, gradually increasing the concentration of buffer B (0.1% formic acid in acetonitrile) from 5% to 35% to effectively separate peptides. Throughout the analysis, the column temperature was maintained at 40 °C with a constant flow rate of 200 nL/min. Ionization was achieved using a 2 kV electrospray voltage relative to the mass spectrometer inlet. The instrument operated in data-independent acquisition (DIA) mode, automatically alternating between full mass spectrometry (MS) and tandem mass spectrometry (MS/MS) modes to enhance analytical comprehensiveness and depth.

The protein qualitative criteria were as follows: precursor threshold 1.0%, FDR; protein threshold, 1.0% FDR. The decoy database was generated using the mutated strategy, which is similar to the shuffled sequence of a random number of amino acids (minimum 2 amino acids, maximum half of the total length of the peptide). Spectronaut performed automatic correction and used a local normalization strategy for data normalization. MaxLFQ was used for protein group quantification for peptides less than 1.0% FDR.

The source of the protein data bank is the Ensembl database (https://asia.ensembl.org/index.html), the use version is Ensembl_release110, and the download date is 28 February 2024.

### 5.5. Bioinformatics Analysis

The workflow leveraged Spectronaut 18 as the core software for DIA data processing, with stringent FDR control (1% at feature levels), dynamic mass time calibration, and MaxLFQ-based quantification. Static (carbamidomethylation) and dynamic (oxidation) modifications were explicitly defined, while mass error tolerance was dynamically optimized using iRT peptides. Downstream analyses employed R packages for statistical testing (5% FDR for DEPs) and bioinformatics tools (Pfam_scan) for functional annotation. All software settings adhered to community standards for reproducibility and analytical rigor in DIA proteomics.

Bioinformatics methods were employed to analyze significantly differential proteins in Hu sheep placentas across gestational stages. First, Gene Ontology (GO) database annotations classified these proteins into biological processes, molecular functions, and cellular components. Kyoto Encyclopedia of Genes and Genomes (KEGG) pathway analysis then identified functionally enriched pathways and genomic interactions critical to placental development (significance thresholds: *p* < 0.05, FDR Q < 0.05). For data visualization, the R programming language and OmicShare online platform (Guangzhou Gidio Biotech, Guangzhou, China) generated heatmaps, enrichment bubble plots, volcano plots, and Venn diagrams. Protein–protein interaction (PPI) networks were constructed using the STRING v11.5 database and visualized via Cytoscape 3.7.1 to elucidate regulatory relationships among differential proteins during placental maturation.

### 5.6. Quantitative Real-Time PCR (qRT-PCR)

Total RNA was extracted from placental tissues using Trizol reagent (TaKaRa Bio Inc., Shiga, Japan, CAS: 9108). Frozen placental samples (0.1 g) were homogenized in sterile, nuclease-free grinding tubes with 1 mL of pre-chilled Trizol reagent and stainless-steel beads. After incubation at room temperature for 5 min, homogenates were centrifuged at 12,000× *g* for 5 min (4 °C). The supernatant was transferred to a new 1.5 mL EP tube, mixed with 200 μL chloroform by vigorous vortexing for 15 s, and incubated on ice for 5 min before centrifugation at 12,000× *g* for 15 min (4 °C). A 400 μL aliquot of the aqueous phase was combined with an equal volume of chilled isopropanol, inverted 15 times, and incubated on ice for 10 min. RNA pellets were collected by centrifugation at 12,000× *g* for 10 min (4 °C), washed twice with 75% ethanol (12,000× *g*, 5 min, 4 °C), and air-dried in a laminar flow hood. RNA was dissolved in RNase-free water and quantified using a Thermo NanoDrop 2000 spectrophotometer (Thermo Scientific, Waltham, MA, USA). RNA integrity was verified by 1.5% agarose gel electrophoresis, with intact RNA exhibiting a 28S:18S ribosomal RNA band intensity ratio of ~2:1 under UV visualization.

For cDNA synthesis, RNA was diluted to 500 ng/μL in nuclease-free water. Reverse transcription was performed using the PrimeScript RT reagent kit (Accurate Biotechnology, Changsha, China Cat NO. AG11728) according to the manufacturer’s protocol. Synthesized cDNA was stored at −20 °C for subsequent qRT-PCR analysis. The primers and amplicon sizes of the genes are shown in Table S1.

### 5.7. Protein Extraction and Western Blot

Total tissue proteins were extracted using a reagent mixture of RIPA lysis buffer, PMSF, and phosphatase inhibitors in a 100:1:1 ratio (*v*/*v*/*v*). Protein concentrations were quantified using a BCA Protein Assay Kit (Beyotime Biotechnology, Beijing, China, Cat. #P0010) according to the manufacturer’s instructions. Samples were diluted to 1 μg/μL, mixed with 20 μL of 5× SDS-PAGE loading buffer, adjusted to a final volume of 100 μL with RNase-free water, and denatured at 100 °C for 5 min.

For Western blot analysis, proteins were separated by SDS-PAGE at 150 V for 45 min and transferred to PVDF membranes at 300 V for 45 min. Membranes were blocked with 5% non-fat milk in TBST buffer for 2 h to minimize nonspecific binding. Primary antibodies (Table S2) (5% dilution in blocking buffer) were incubated with the membranes at 4 °C for 12–16 h, followed by three 10 min TBST washes. Horseradish peroxidase (HRP)-conjugated secondary antibodies (5% dilution in 5% non-fat milk/TBST) were applied for 1 h at room temperature, with subsequent TBST washes. Membranes were incubated in ECL reagent for 1 min and imaged using a high-sensitivity chemiluminescence detection system. Band intensities were quantified using ImageJ v. 1.53t software to assess target protein expression levels.

### 5.8. Statistical Analysis

All experimental results are presented as mean ± standard error of the mean (SEM). Data were subjected to one-way analysis of variance (ANOVA) using SPSS software (version 24.0, IBM Corporation, Armonk, NY, USA). Significance thresholds were defined as follows: *p* < 0.05 (significant) and * *p* < 0.01 (highly significant). 

Protein differential expression was statistically evaluated using a two-tailed Student’s *t*-test with Benjamini–Hochberg false discovery rate (FDR) correction. Significant candidates were identified through dual-threshold filtering: (1) magnitude thresholds of >1.2-fold upregulation or <0.83-fold downregulation (log_2_ ratio >0.26 or <−0.26) and (2) significance threshold of FDR-adjusted *p*-value < 0.05.

## Data Availability

All the data generated or analyzed in this study are included in this paper. DIA proteomic sequence data are available in the ProteomeXchange database with IPX0011294001.

## Supplementary Materials

The following supporting information can be downloaded at: https://www.mdpi.com/article/10.3390/ijms26094236/s1.
