# Supplementary material for: Proteomic Profiling of Hu Sheep Placental Development Across Gestational Stages Reveals Stage-Specific Regulatory Networks
Source: Int J Mol Sci. 2025 Apr 29;26(9):4236. doi: 10.3390/ijms26094236 (PMC12071506; doi:10.3390/ijms26094236)
Supplement: Supplementary file 1 [file ijms-26-04236-s001.zip › Supplementary material.pdf]

Table S1

Gene		Primers (5'-3')	Bp	Accession
OCLN	Forward	GCCTGTGTTGCCTCCACTCTTG	131	>XM_015101255.3
	Reverse	CCGTAGCCATAACCATAGCCATAGC		
CLDN-1	Forward	GTCTTTGGGGGCGTGATCTT	150	>NM_001185016.1
	Reverse	CCAGCCAATGAAGAGAGCCT		
CLDN-4	Forward	CACGCAACAACAAGCCCTAC	288	>NM_001185017.2
	Reverse	GTGGCCCAGGAGTCTCTTTC		
PCNA	Forward	AGTGGCGTGAACCTACAGAG	203	>XM_004014340.3
	Reverse	GCCAAGGTGTCCGCATTATC		
BAX	Forward	CGAGTGGCGGCTGAAAT	286	>XM_015100640.1
	Reverse	GGTCTGCCATGTGGGTGTC		
Bcl2	Forward	CGCATCGTGGCCTTCTTT	113	>XM_012103831.2
	Reverse	CGGTTCAGGTACTCGGTCATC		
Caspase3	Forward	ATAGCGCAGGGAACTACAGTC	112	>XM_060406953.1
	Reverse	GTTTCCGGTGCATAGCAAGTG		
Caspase9	Forward	GGCGGTGGAGGTGAGATG	162	>XM_060396599.1
	Reverse	AAGAAGAGCTTGGGCTTCCC		
VEGFA	Forward	ACCAAAGCCAGCACATAGGA	102	>NM_001025110.1
	Reverse	TCGGCTTGTCACATTTTTCTTGC		
PGF	Forward	CAGTCTGTGCCTCCTCCCTAGC	123	>XM_060418672.1
	Reverse	CTGGCTCCCTCTGTCCCTGTG		
GCM-1	Forward	GCCAGCCATCTGCGACAAGG	134	>XM_042237025.2
	Reverse	GTCCATCGTGCCTCCAGAAGTTG		
Syncytin	Forward	ACCTCACCGACACTACCGTTACC	107	>XM_060408712.1
	Reverse	CTAAGCAGGCACCGGGTTACTTC		
SLC2A1	Forward	TGGGAAAGTCCTTTGAGATGC	107	>NM_174602.2
	Reverse	GGTCAGGCCGCAGTACACA		
SLC2A3	Forward	ATCTGGCTGCCTTTCCTTGT	208	>NM_001009770.1
	Reverse	CTCTGGCCACCCCAGATCTAT		
TJP1	Forward	CGACCAGATCCTCAGGGTAA	161	>XM_060401403.1
	Reverse	AATCACCCACATCGGATTCT		
TJP2	Forward	CAGCGTTCAGAATGCCCAGAGAG	121	>XM_027964513.2
	Reverse	GGCGGCGGTCAGGTCCTC		
SLC7A1	Forward	TCGGCACTCTCCTGGCTTACTC	150	>XM_042254846.1
	Reverse	AATCGCTGCTGCTCACCAACTC		
CLND7	Forward	GCAGAGCACCGGCATGATGAG	103	>NM_001185018.1
	Reverse	CAGCACCAGGGAAACCACCATTAG		
ACTB	Forward	CGCAAGTACTCCGTGTGGAT	146	>NM_001009784.3
	Reverse	TAACGCAGCTAACAGTCCGC		
MAP3K1	Forward	GCGCTTACGCTGGTCTTCTC	271	XM_060400255.1
	Reverse	CGAGAAGGCTGGGCTAGATTT		
CLND8	Forward	AGGCAGCCAGAGGACTGATGTG	138	>XM_004002779.5
	Reverse	CTCCAGCGGTCAGCAGAATGTG		

Table S2

Antibodies	Cat No.	Source	Dilution of WB
PCNA	AF0239	Affinity	1:1,000
BAX	50599-2-lg	Proteintech	1:5,000
Vinculin	66305-1-lg	Proteintech	1:5,000
ERK	A4782	Abconal	1： 1000
p-ERK	af1891	碧云天	1： 1000
